# Effect of Carboxylic Functional Group Functionalized on Carbon Nanotubes Surface on the Removal of Lead from Water

**DOI:** 10.1155/2010/603978

**Published:** 2011-02-08

**Authors:** Muataz Ali Atieh, Omer Yehya Bakather, Bassam Al-Tawbini, Alaadin A. Bukhari, Faraj Ahmad Abuilaiwi, Mohamed B. Fettouhi

**Affiliations:** ^1^Chemical Engineering Department, King Fahd University of Petroleum & Minerals, Dhahran 31261, Saudi Arabia; ^2^Center of Research Excellences in Nanotechnology, King Fahd University of Petroleum & Minerals, Dhahran 31261, Saudi Arabia; ^3^Earth Sciences Department, King Fahd University of Petroleum & Minerals, Dhahran 31261, Saudi Arabia; ^4^Research Institute, King Fahd University of Petroleum & Minerals, Dhahran 31261, Saudi Arabia; ^5^Hafr Al-Batin Community College, King Fahd University of Petroleum & Minerals, Hafr Al-Batin 31991, Saudi Arabia; ^6^Chemistry Department, King Fahd University of Petroleum & Minerals, Dhahran 31261, Saudi Arabia

## Abstract

The adsorption mechanism of the removal of lead from water by using carboxylic functional group (COOH) functionalized on the surface of carbon nanotubes was investigated. Four independent variables including pH, CNTs dosage, contact time, and agitation speed were carried out to determine the influence of these parameters on the adsorption capacity of the lead from water. The morphology of the synthesized multiwall carbon nanotubes (MWCNTs) was characterized by using field emission scanning electron microscopy (FESEM) and transmission electron microscopy (TEM) in order to measure the diameter and the length of the CNTs. The diameters of the carbon nanotubes were varied from 20 to 40 nm with average diameter at 24 nm and 10 micrometer in length. Results of the study showed that 100% of lead was removed by using COOH-MCNTs at pH 7, 150 rpm, and 2 hours. These high removal efficiencies were likely attributed to the strong affinity of lead to the physical and chemical properties of the CNTs. The adsorption isotherms plots were well fitted with experimental data.

## 1. Introduction

The pollution of water resources due to the disposal of heavy metals has been causing worldwide concern. The main sources of these metals are mining, metallurgical, chemical manufacturing, tannery, battery manufacturing industries, fossil fuel, the modern chemical industry based largely on catalysts, many of which are metals or metal compounds, production of plastics, such as polyvinyl chloride, involving the use of metal compounds, particularly as heat stabilizers, and so forth. The effects of heavy metals such as copper, lead, zinc, mercury, chromium, and cadmium on human health have been investigated extensively. Lead is ubiquitous in the environment and is hazardous at high levels. Long-term drinking water containing high level of lead will cause nervous system damage, renal kidney disease, mental retardation, cancer, and anaemia [1]. Lead is non-biodegradable and, therefore, must be removed from water [2]. Many methods have been developed and used to remove metal ions from wastewater such as granulated activated Carbon [3, 4], fly ash [5], peat [6], recycled alum sludge [7], peanut hulls [8], resins [9], kaolinite [10], manganese oxides [11], zeolite [12], and biomaterials [13, 14]. However, the removal efficiencies of metal ions of these adsorbents were low. Therefore, researchers carried out to evaluate new promising adsorbents [15]. Carbon nanotubes (CNTs), a member in carbon family, have novel properties that make them potentially useful in many applications in nanotechnology, electronics, optics, water treatment, and other fields of materials science. Since their discovery in 1991 [16], carbon nanotubes (CNTs) have attracted considerable researchers' interest due to their exceptional mechanical electrical properties, highly chemical stability, and large specific area [17]. Carbon nanotubes (CNTs) are new adsorbents of trace pollutants from water, because they have a large specific surface area and small, hollow, and layered structures. Multiwalled carbon nanotubes (MWCNT) were previously used for removal of metal ions, such as lead, copper, cadmium, silver, and nickel. Li et al. [18] reported that carbon nanotubes have high adsorption efficiency for lead removal from water and the adsorption of lead is higher than copper and cadmium and significantly influenced by pH. Hsieh and Horng [19] reported that the adsorption capacity of CNTs grown on aluminum oxide for Pb^2+^, Cu^2+^, and Cd^2+^ from solutions is superior to that of active carbon powders, commercial CNTs, and aluminum oxide. Kandah and Meunier [20] found that the adsorption of Ni^2+^ by oxidized CNTs is 1.24 times greater than commercial activated carbon called MINOTAUR. Xu et al. [21] reported that removal of Pb^2+*‏*^ is strongly dependent on pH, ions strength, and the type of foreign ions [22].

The oxidation of carbon surfaces is known to generate not only more hydrophilic surface structures but also more oxygen containing functional groups and to increase the ion-exchange capacity [23]. The specific surface area and pore specific volume of CNTs increase after oxidation with HNO_3_. Their particle sizes decrease due to the fracture where defects exit [24]. The first type of functionalization typically involves oxidation using acids or oxidants, causing carboxyl groups to functionalize the defects and the ends of the CNTs [25]. The adsorption capacity onto the surface of CNTs increased with the pH of the system from acidic to alkaline. pH is one of the most important factors that affect the sites of dissociation of CNTs and the hydrolysis, complexation, and precipitation of metal ions. At acidic pH, the surface is expected to have a net positive charge and to adsorb few Pb^2+^ ions. Above pH, a net negative charge is present on the surface of the CNTs, promoting the adsorption of Pb^2+^, because of the electrostatic force of attraction between Pb^2+^ and the surface of the CNTs. The Pb^2+^ adsorption capacity rapidly increased with pH above 8.5, which in fact can be explained by the precipitation of Pb^2+^from solution [26]. The dependence of adsorption on pH is associated with the dependence of the surface charge on CNTs on pH [27]. 

In this work the effect of modified and nonmodified carbon nanotubes was used to study the effect of these nanomaterials on the removal of the lead from water. The process parameters such as pH, dosage of MWCNTs, agitation speed, and contact time were investigated in order to maximize the removal of Pb^2+^. 

## 2. Experimental

### 2.1. Production of Carbon Nanotubes

The experimental set-up used to synthesize the Multiwall Carbon Nanotubes is similar to that reported by Muataz et al. [28, 29]. The Floating Catalyst Chemical Vapor Deposition (FC-CVD) reactor has been used to produce CNTs. The production of CNTs in the present work has been conducted in a horizontal tubular reactor. The horizontal reactor is a quartz tube of 50 mm in diameter and 900 mm in length and heat by silicon carbide heating element. In this study benzene (C_6_H_6_ 99.5% purity) was used as a hydrocarbon source and ferrocene (FeC_10_H_10_ 98% purity) and Hydrogen as carrier gas and argon for flushing the air from the system. Several experiments were performed at the reaction temperature ranging from 500°C to 1200°C. Other conditions like the reaction time (45 minutes) and hydrogen flow rate (300 mL/min) were fixed. The produced carbon nanotubes were characterized by using Field emission scanning electron Microscopy (FE-SEM) and Transmission Electron Microscopy (TEM). 

### 2.2. Oxidation of MWCNTs

**Figure 1:**
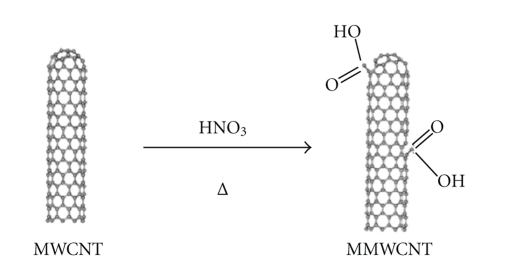
Chemical modification of carbon nanotubes (MWCNTs) through thermal oxidation.

Multiwall Carbon Nanotubes (MWCNTs) were used in this study. The Purity of MWCNT is >95%, its outside and inside diameters are 10–20 nm and 5–10 nm, respectively, and their length reaches up to 10–30 *μ*m. Three hundred milliliters of a concentrated nitric acid of AnalaR (69%) are added to 2 g of as-received MWCNT. The mixture is refluxed for 48 h at 120°C. After cooling at room temperature, the reaction mixture is diluted with 500 ml of deionized water and then vacuum-filtered through a filter paper (3 *μ*m porosity). This washing operation is repeated until the pH became the same as deionized water pH and is followed by drying in a vacuum oven at 100°C. Such conditions lead to remove the catalysts from carbon nanotubes and opening the tube caps as well as the formation of holes in the sidewalls, followed by an oxidative etching along the walls with the concomitant release of carbon dioxide. This less vigorous condition minimized the shortening of the tubes and the chemical modification is then limited mostly to the opening of the tube caps and the formation of functional groups at defect sites along the sidewalls. The final products are nanotube fragments whose ends and sidewalls are decorated with a various oxygen containing groups (mainly carboxyl groups) (Figure 1). Moreover, the percentage of carboxylic functions on the oxidized MWCNT surface does not exceed 4% in the best cases, which corresponds to the percentage of MWCNT structural defects [30–33]. 

### 2.3. Preparing the Stock Solution

The stock solution was prepared by adding 2 mL of lead from lead standard solution of concentration 1000 mg/L into 2 L volumetric flask. The pH of the stock solution was adjusted by using 1.0 M Nitric Acid or 1.0 M NaOH. Finally buffer solutions were added to maintain the pH constant during the experimental.

### 2.4. Batch Mode Adsorption Experiment

The experiment of the batch mode adsorption was conducted at room temperature to study the effect of initial solution pH, CNTs dosage, contact time, and agitation speed on the adsorption of Pb^2+*‏*^ ions. Each experiment was conducted in volumetric flask and the initial and final concentrations of Pb^2+^ *‏* were analyzed by using Inductively Coupled Plasma (ICP). 

## 3. Results and Discussion

### 3.1. Characterization of Carbon Nanotubes

**Figure 2:**
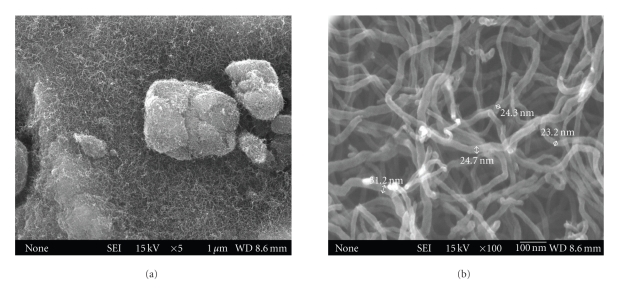
SEM Images of carbon nanotubes (a) at low resolution and (b) at high resolution.

Multiwall carbon nanotubes were produced by chemical vapor deposition (CVD) technique. The produced carbon nanotubes were characterized by using field emission scanning electron microscopy (FE-SEM) and transmission electron microscopy (TEM). The diameters of the produced carbon nanotubes varied from 20 to 40 nm with average diameter at 24 nm while the length of the CNTs was up to few microns.  Figure 2(a) shows the SEM image of carbon nanotubes at low magnification while Figure 2(b) shows the SEM image of carbon nanotubes at high magnification. From the SEM observation, the product is pure and only carbon nanotubes were observed.

**Figure 3:**
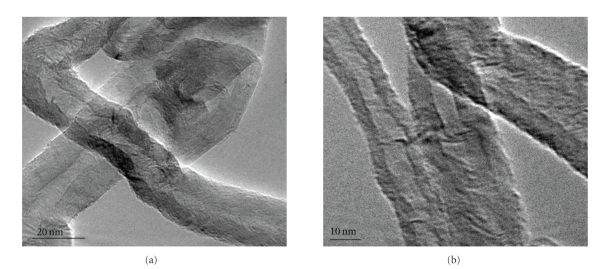
TEM Images of carbon nanotubes (a) at low resolution and (b) at high resolution.

TEM was carried out to characterize the structure of nanotubes (Figure 3). To prepare TEM samples, some alcohol was dropped on the nanotubes film, and then, these films were transferred with a pair of tweezers to a carbon-coated copper grid. It is obvious from the images that all the nanotubes are hollow and tubular in shape. In some of the images, catalyst particles can be seen inside the nanotubes. TEM images indicate that the nanotubes are of high purity, with uniform diameter distribution, and contain no deformity in the structure while Figure 3(b) shows the High-Resolution Transmission Electron Microscope (HRTEM) of the carbon nanotubes. It shows that a highly ordered crystalline structure of CNT is present.

### 3.2. Functionalization of CNTs with Carboxylic Functional Group (COOH)

**Figure 4:**
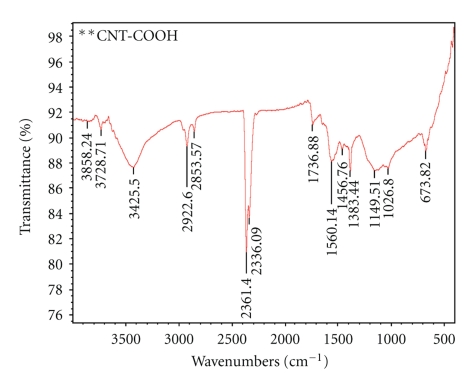
FTIR of Carbon Nanotubes (CNTs) modified with COOH.

FTIR spectra from the MWCNTs show a broad peak at *∼*3425 cm^−1^ which is a characteristic of the O-H stretch of hydroxyl group (Figure 4) which can be ascribed to the oscillation of carboxyl groups. Carboxyl group on the surface of MWCNTs could be due to the partial oxidation of the surface of MWCNTs during purification by the manufacturer. This feature moves to 1736 cm^−1^, associated with the stretch mode of carboxylic groups as observed in the IR spectrum of the acid-treated MMWNTs indicating that carboxylic groups are formed due to the oxidation of some carbon atoms on the surface of the MWNTs by nitric acid. The IR spectra of oxidized MWCNTs show four major peaks, located at 3728, 3425, 2361, and 1560 cm^−1^. The peak at 3728 cm^−1^ is attributed to free hydroxyl groups. The peak at 3425 cm^−1^ can be assigned to the O–H stretch from carboxyl groups (O=C−OH and C−OH) while the peak at 2361 cm^−1^ can be associated with the O−H stretch from strongly hydrogen-bonded −COOH. The peak at 1560 cm^−1^ is related to the carboxylate anion stretch mode. It should be noticed that the as-received MWCNTs were purified by the manufacturer and part of the catalytic metallic nanoparticles was possibly eliminated during the purification process cutting the nanotube cap. Thus, the presence of carboxylic groups in these MWCNTs can be expected [32, 33].

### 3.3. Effect of pH

**Figure 5:**
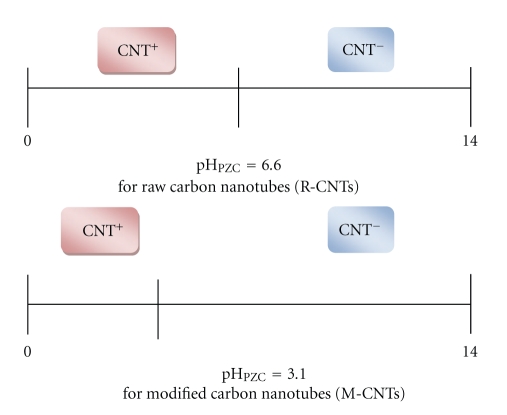
Point of zero charge of modified and nonmodified Carbon Nanotubes.

The pH of aqueous solution is an important variable, which controls the adsorption of ion at the solid-water interfaces. The pH is also said to be an important parameter for the adsorption of metal ions from aqueous solution because it affects the solubility of the metal ions, concentration of the counter ions on the functional groups of adsorbent, and the degree of ionization of the adsorbate during the reaction. When pH of the solution is higher than pH_PZC_ (Point of Zero Charge), the negative charge on the surface provides electrostatic interactions that are favorable for adsorbing cationic species. It has been found that the point of zero charge “pH_PZC_” of as-received raw Carbon Nanotubes is 6.6 while for the M-CNTs with carboxylic functional group shift the pH_PZC_ to 3.1 ( as shown in Figure 5) [34]. 

**Figure 6:**
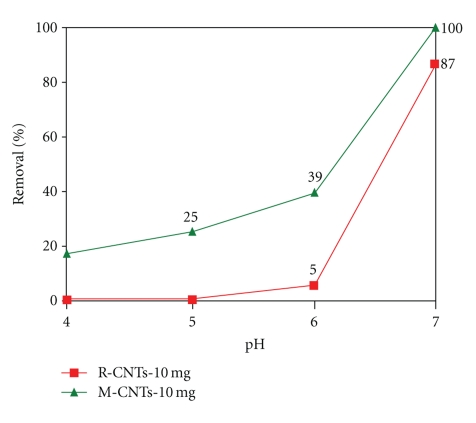
The effect of pH on percentage removal of lead at 150 rpm.

The decrease of pH leads to neutralization of surface charg; thus, the adsorption of cations should decrease. The pH value plays a major role with respect to the adsorption of Pb^2+^ ions on CNTs. The removal of lead by two types of adsorbents (modified and nonmodified CNTs) with various pH has been studied. The pH of these experiments varied from 3 to 7. Precipitation will occur between Pb^2+^ *‏* and OH^−^ as the pH exceeds pH 7.0 Li et al. [35] To avoid the conflict in the result for the removal of Pb^2+^ by either CNTs or precipitation, our experiments were carried out only under these conditions.  Figure 6 shows the effect of pH on the adsorption of Pb^2+^, which was used as a model of divalent metal ion on raw carbon nanotubes (R-CNTs) and M-CNTs carbon nanotubes (M-CNTs). The obtained results indicate that the functional groups introduced by oxidation increased the ion-exchange capacities of carbon nanotubes and make Pb^2+^ adsorption capacity increase correspondingly. The adsorption of Pb^2+^ species increased with the increase of pH from 3 to 7, but more sharp increase was observed for oxidized CNTs because of the chemical interaction between the metal ions and the surface functional groups such as hydroxyl (–OH), carboxyl (–COOH), and carbonyl (–C=O). These functional groups attached on the surfaces of the CNTs improve their adsorption capability of Pb^2+^ in solution. The low adsorption that took place in acidic region (pH 4-5) can be attributed in part to competition between H^+^ and Pb^2+^ ions on the same sites. Furthermore, the charge of CNTs surface becomes more negative with the increase of pH, which causes electrostatic interactions and thus results in higher adsorption of metal species. The result shows that the adsorption of lead increases with an increase on the pH of the solution from 4 to 7 by using both R-CNTs and M-CNTs. The maximum removal of Pb^2+^ *‏* by using R-CNTs was 87 percent at pH 7, while the removal of lead at pH 4 and 5 was zero because at acidic region, the surface of the raw carbon nanotubes becomes more neutralized and no negative charge provides electrostatic interactions that are favorable for adsorbing of Pb^2+^ species. The maximum removal of lead by using M-CNTs was 100 percent at pH 7. At low pH (4 and 5), the removal of lead from the solution by using M-CNTs was 17 and 25 percent, respectively. It was reported that at the acidic region there would be a strong competition between the H^+^ and Pb^2+^, which will reduce the removal capacity of lead by the functional group. It can be observed that the removal of lead from water by using M-CNTs is tremendous higher than the raw carbon nanotubes due to the ionization step by the functional group on the surface of CNTs. 

This study suggests that modifying the surface of CNTs not only can make it more negatively charged and hydrophilic but can also form various functional groups, substantially promoting the adsorption of Pb^2+^ onto modified CNTs. The functional groups by acid/oxidation improved the ion-exchange capabilities of the CNTs and increased Pb^2+^ adsorption capacities correspondingly.

### 3.4. Effect of Contact Time

**Figure 7:**
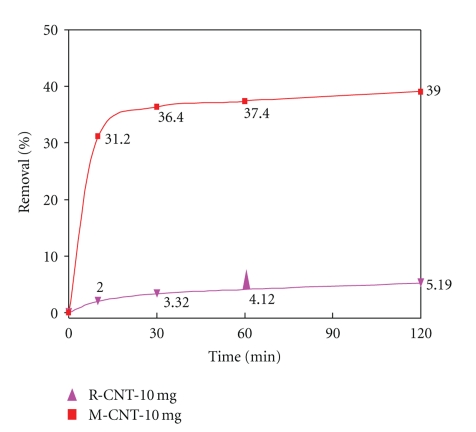
The effect of time on percentage removal of lead at 150 rpm pH 6.

By keeping the carbon nanotubes dosage, agitation speed, and pH at constant values, it was observed that lead adsorption has positive result in terms of time.  Figure 7 shows that the amount of Pb^2+^ *‏* adsorbed onto both raw carbon nanotubes (R-CNTs) and modified CNTs (M-CNTs) increased rapidly during the beginning 10 minutes. Subsequently, the adsorption rate rises gradually and reaches equilibrium after 30 and 60 minutes for Pb^2+^ *‏* adsorption by using (M-CNTs) and (R-CNTs), respectively. It has been observed that there will be slightly increase on the removal of Pb^2+^ *‏* after 60 min for the R-CNTs. The short time required to reach equilibrium suggests that the M-CNTs have very high adsorption capacity for the Pb^2+^ concentration of the test and have a great potential in Pb^2+^ adsorbent application.

### 3.5. Effect of Agitation Speed

**Figure 8:**
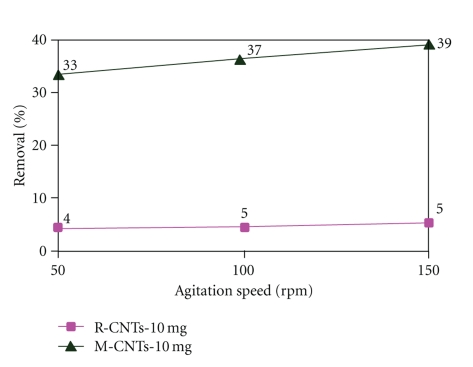
The effect of agitation speed on percentage removal of lead at pH 6.

The effect of agitation speed on adsorption capacity of lead has been studied by varying the speed of agitation from 50 to 150 rpm (as shown in Figure 8). It has been observed that the percentage of lead removal increased slightly by increasing agitation speed. This is due to the fact that the increase of agitation speed improves the diffusion of lead ions towards the surface of the adsorbents and decreases the mass transfer resistance when agitation increases and offers a faster external mass transfer rate of Pb^2+^ and thus gives more adsorption capacity.

### 3.6. Effect of CNTs Dosage

**Figure 9:**
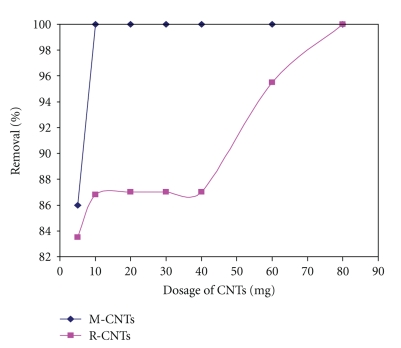
The effect of dosage of CNTs on percentage removal of lead at 150 rpm at pH 7.

**Figure 10:**
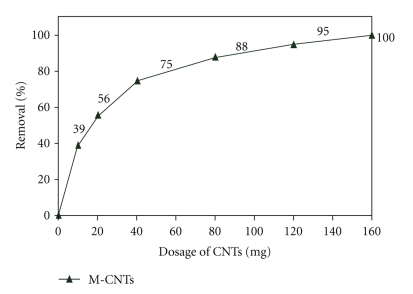
The effect of dosage of CNTs on percentage removal of lead at 150 rpm at pH 6.

The batch adsorption experiments were carried out by using various amounts of R-CNTs and M-CNTs from 5 to 80 mg while the pH, agitation speed, and contact time were fixed at 7, 150 rpm, and 120 min, respectively. It has been noted that by increasing the amount of CNTs into the solution the removal of lead increased. By using M-CNTs the removal reaches up to 100 percent by adding 10 mg while by using R-CNTs the maximum removal of lead was 100 percent after adding 80 mg of adsorbent which could be due to the availability of more sorption sites (as shown in Figure 9). Apart from that, more experimental studies were carried out by using M-CNTs at pH 6 to find the optimum dosage since the maximum removal of lead at pH 7 was 100 at 10 mg. It was found that the maximum removal of lead was 100 percent when 160 mg of M-CNTs were added (as shown in Figure 10). Up to a certain value, no further increase in percent sorption of metal ion occurred as an increase in CNT mass.

### 3.7. Freundlich and Langmuir Isotherms Models

Adsorption isotherms are mathematical models that describe the distribution of the absorbate species between liquid and adsorbent, based on a set of assumptions that are mainly related to the heterogeneity/homogeneity of adsorbents, the type of coverage, and possibility of interaction between the adsorbate species. The Langmuir model assumes that there is no interaction between the adsorbate molecules and the adsorption is localized in a monolayer. The Freundlich isotherm model is an empirical relationship describing the adsorption of solutes from a liquid to a solid surface and assumes that different sites with several adsorption energies are involved.

In totality, Freundlich and Langmuir isotherms relate the coverage or adsorption of molecules on a solid surface to gas pressure or concentration of a medium above the solid surface at a fixed temperature. The experimental data for Pb^2+^ adsorption on CNTs at different pH values could be approximated by the isotherm models of Langmuir (1) and Freundlich (2):

(1) $$q=\frac{q_{m}K_{L}C}{1+K_{L}C\;},$$

where *C* is the equilibrium lead concentration (mg/l), *q* is the amount adsorbed (mg/g), and *q*_*m*_ and *K*_*L*_ are Langmuir constants related to adsorption capacity and energy of adsorption, respectively:

(2) $$q=K_{F}c^{1/n},\;$$

where *K*_*F*_ and *n* are Freundlich constants related to adsorption capacity and adsorption intensity, respectively.

Equations (1) and (2) can be written as

(3) $$\frac{C}{q}=\frac{1}{\left(K_{L}q_{m}\right)}+\frac{C}{q_{m}},$$

(4) $$\log\;\;q=\frac{1}{n}\log\;\;C+\log\;\;K_{F}.$$

#### 3.7.1. Langmuir and Freundlich Adsorption Isotherm Models for Lead

**Figure 11:**
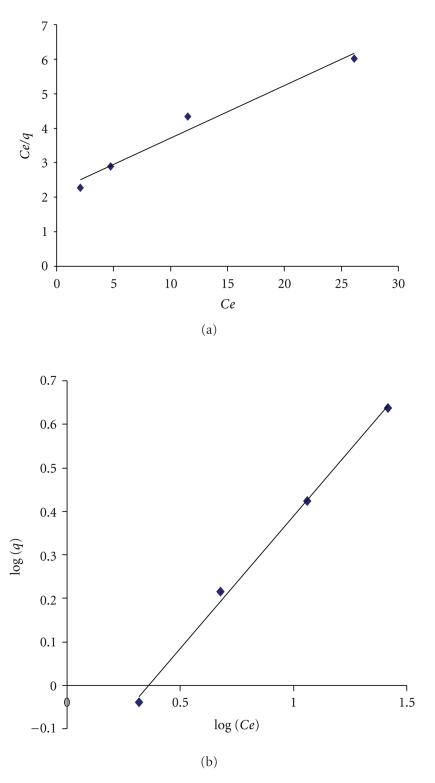
Adsorption isotherm model for lead (a) Langmuir and (b) Freundlich.

It can be seen from Table 1 that both Langmuir and Freundlich models show good agreement with the experimental data, with the correlation coefficient values of 0.9731 and 0.9971, respectively.  Figure 11 presents the linear, Langmuir and Freundlich isotherm plots of Pb^2+*‏*^ adsorption on the M-CNTs at pH 6 because R-CNTs shows low adsorption rate of Pb^2+^ *‏* at low dosage of CNTs. The equilibrium data were fitted very well to both sorption isotherms. Therefore, this indicates the applicability of monolayer coverage of Pb^2+*‏*^ ions on the surface of the adsorbent. This is due to the fact that CNTs have greater surface area for metal adsorption. The good correlation coefficient of Langmuir and Freundlich isotherm also indicates that Pb^2+*‏*^ ions adsorbed to the surface of M-CNTs. Therefore, it is verified that CNTs have great potential to be a good adsorbent for the removal of Pb^2+*‏*^ ions in water treatment [22].

### 3.8. Modeling of Kinetics Adsorption

Modeling of kinetic data is fundamental for the industrial application of adsorption since it gives information for comparison among different biomaterials under different operational conditions for designing and optimizing operational conditions for pollutant removal from wastewater systems [22].

The study of sorption kinetics is applied to describe the adsorbate uptake rate and this rate evidently controls the residence time of adsorbate at solid liquid interface. In order to evaluate the mechanism of sorption of Pb^2+^ by the CNTs, the first-order equation, the pseudo-second-order rate equation, and the second-order rate equation are calculated by the following equations, respectively:

(5) $$\log\;\;\frac{q_{e}-q_{t}}{q_{e}}=-\frac{K_{L}t}{2.303},$$

(6) $$\frac{t}{q_{t}}=\;\frac{1}{2K_{s}q_{e}^{2}}+\;\frac{t}{q_{e}},$$

(7) $$\frac{1}{q_{e}-q_{t}}=\;\frac{1}{q_{e}}+\;kt,$$

where *q*_*e*_ is the orption capacity at equilibrium, *q*_*t*_ is the sorption capacity at time (mg/g), *K*_*L*_ is the Lagergren rate constant of adsorption (1/min), *k* is the rate constant of the pseudo-second-order sorption (g·mg^−1^·min^−1^), and *t* is the time (min).

The linear plots of log (*q*_*e*_ − *q*_*t*_) versus *t*, *t*/*q*_*t*_ versus *t*, and 1/(*q*_*e*_ − *q*_*t*_) versus *t* of the above equations, and *q*_*e*_, *K*_*L*_, and *k* can be determined from the slopes and intercepts. 

The kinetics was investigated by using the information obtained from the effect of dosage (dry-weight basis) at 25°C at three different time intervals up to 120 min. The pseudo-first-order kinetic equation was not applicable because *R* ^2^ is small comparing to *R* ^2^ of pseudo-second-order equation. Therefore, the pseudo-second-order equation was used in this study in order to investigate the mechanism of adsorption of lead by the CNTs and the potential rate-controlling steps, such as mass transport and chemical reactions:

(8) $$\frac{dq_{t}}{dt}=K_{2}{\left(q_{e}-\;q_{t}\right)}^{2},$$

where *q*_*e*_ and *q*_*t*_ are the sorption capacity at equilibrium and at time (mg/g), respectively, and K^2^ is the rate constant of the pseudo-second-order sorption (g·mg^−1^·min^−1^). For the boundary conditions *t* = 0 to *t* = *t* and *q*_*t*_ = 0 and *q*_*t*_ = *q*_*t*_, the integrated form of (8) becomes

(9) $$\frac{1}{q_{e}-q_{t}}=\frac{1}{q_{e}}+k_{2}t.$$

This has a linear form:

(10) $$\frac{1}{q_{t}}=\frac{1}{K_{2}q_{e}^{2}}+\frac{t}{q_{e}}.$$

The integrated form of the equation is

(11) $$\frac{t}{q_{t}}=\frac{1}{h}+\left(\frac{1}{q_{e}}\right)t,$$

where *h* (g·mg^−1^·min^−1^) can be regarded as the initial sorption rate *q*_*t*_/*t* → 0; hence

(12) $$h=k_{2}{q_{e}}^{2}.$$

If the pseudo-second-order kinetics is applicable to the experimental data, the plot of *t*/*q*_*e*_ versus time of (11) gives a linear relationship from which *q*_*e*_, *k*, and *h* can be determined from the slope and intercept of the plot, respectively.

### 3.9. Kinetics Adsorption Model of Lead

The kinetics adsorption model has been done for lead at pH 6 instead of pH 7 to avoid the conflict the results because of possibility of precipitation of lead at pH 7. The parameters of modeling are shown in Table 2.

**Figure 12:**
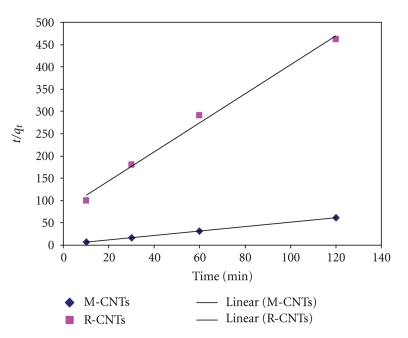
Pseudo second-order kinetics of Pb^2+^ *‏* using R-CNTs and M-CNTs.

The plot of *t*/*q*_*t*_ versus time (Figure 12) yields very good straight lines (correlation coefficient, *R* ^2^ = 0.9934 for R-CNTs and *R* ^2^ = 0.9998 for M-CNTs). The second-order rate constant obtained from this (Figure 12) is 8.0439 for R-CNTs and 9.7273 (g·mg^−1^·h^−1^) for M-CNTs. The second-order rate constant indicates that time to achieve equilibrium concentration of Pb^2+*‏*^ is less by using M-CNTs compared with R-CNTs. The equilibrium adsorption capacity *q*_*e*_ obtained from the graph also implies that M-CNTs have higher adsorption capacity (*q*_*e*_ = 2 mg/g) compared to R-CNTs (*q*_*e*_ = 0.30672 mg/g). However, the pseudo-first-order model did not provide as good a fit to the data (*R* ^2^ < 0.9).

## 4. Conclusion

Carbon Nanotubes were found to be efficient for the adsorption of Pb^2+^ *‏* in aqueous solution. The characterization of Pb^2+*‏*^ uptake showed that the lead binding is dependent on initial pH, agitation speed, amount of dosage, and contact time. Percentage uptake increased with an increased in pH from pH 4 to pH 7. The optimum pH found in this study is pH 7 in which it gave 87% removal of Pb^2+^ *‏* ions by using R-CNTs and 100% of Pb^2+*‏*^ ions by using M-CNTs from aqueous solution. The percentage uptake increases slightly with an increase in agitation speed from 50 to 150 rpm, in which 150 rpm gave slightly higher removal for lead, while the percent removal of Pb^2+*‏*^ was observed to be optimal for higher dosage of CNTs, in which 10 mg of M-CNTs contribute to 100% removal of Pb^2+^ *‏*.

## Figures and Tables

**Table 1:** Parameters of Langmuir and Freundlich adsorption isotherm models for lead.

Langmuir			Freundlich		
*q*_*m*_	*K*_*L*_	*R* ^2^	*n*	*K*_*F*_	*R* ^2^
6.6	0.0704	0.9731	1.6437	0.6041	0.9971

**Table 2:** Kinetic parameters for pseudo-second-order model of lead.

Adsorbent (10 mg)	*q*_*e*_ (mg/g)	*K*_2_ (g·mg^−1^·h^−1^)	*R* ^2^
R-CNTs	0.30672	8.0439	0.9934
M-CNTs	2	9.7273	0.9998
