# Corrigendum to “Effect of Carboxylic Functional Group Functionalized on Carbon Nanotubes Surface on the Removal of Lead from Water”

**DOI:** 10.1155/2018/2687413

**Published:** 2018-10-22

**Authors:** Muataz Ali Atieh, Omer Yehya Bakather, Bassam Tawabini, Alaadin A. Bukhari, Faraj Ahmad Abuilaiwi, Mohamed B. Fettouhi

**Affiliations:** ^1^Chemical Engineering Department, King Fahd University of Petroleum & Minerals, Dhahran 31261, Saudi Arabia; ^2^Center of Research Excellences in Nanotechnology, King Fahd University of Petroleum & Minerals, Dhahran 31261, Saudi Arabia; ^3^Earth Sciences Department, King Fahd University of Petroleum & Minerals, Dhahran 31261, Saudi Arabia; ^4^Research Institute, King Fahd University of Petroleum & Minerals, Dhahran 31261, Saudi Arabia; ^5^Hafr Al-Batin Community College, King Fahd University of Petroleum & Minerals, Hafr Al-Batin 31991, Saudi Arabia; ^6^Chemistry Department, King Fahd University of Petroleum & Minerals, Dhahran 31261, Saudi Arabia

In the article titled “Effect of Carboxylic Functional Group Functionalized on Carbon Nanotubes Surface on the Removal of Lead from Water” [1], the name of the third author was given incorrectly as Bassam Al-Tawbini. The author's name should have been written as Bassam Tawabini. The revised authors' list is shown above.
